# Oral health of cystic fibrosis patients at a north american center: A pilot study

**DOI:** 10.4317/medoral.22756

**Published:** 2019-04-24

**Authors:** Raya Abu-Zahra, Nicholas J. Antos, Theresa Kump, Matina V. Angelopoulou

**Affiliations:** 1 Pediatric Dentist, former Resident at Children’s Hospital of Wisconsin, Milwaukee, WI; 2 Assistant Professor Medical College of Wisconsin, Milwaukee, WI; 3 Clinical Research Manager, Medical College of Wisconsin, Milwaukee WI; 4 Assistant Professor Marquette University, School of Dentistry, Milwaukee, WI

## Abstract

**Background:**

The objective of this study was to describe the oral health status of Cystic Fibrosis (CF) children in a US facility.

**Material and Methods:**

Twenty CF children ages 6-18 were recruited from Children’s Hospital of Wisconsin Pulmonary Clinic. Parents completed a health questionnaire. Clinical examinations checked dental caries using the dmft/DMFT index, dental hygiene using the Simplified Greene-Vermillion Index (DI-S), gingival inflammation using the Community Periodontal Index of Treatment Needs, and enamel defects using the modified Developmental Defects of Enamel Index.

**Results:**

The majority (90%) brush twice a day, 65% consume sugary snacks, and 70% visit the dentist every 6 months. Clinically, they presented DMFT 0.25 and dmft 0.90, fair oral hygiene with DI-S 1.02, 75% had mild gingivitis and 50% had enamel defects. The more antibiotics they took, significantly more frequent (*p*=0.007) and more severe (*p*=0.017) enamel defects were noted. Similar trend was found between the number of surgeries and the presence of enamel defects (*p*=0.076) and dental caries (*p*=0.028).

**Conclusions:**

Within the limitations of this study, CF patients were found to be at oral health risk due to the high prevalence of dental enamel defects. Oral health for CF children should be part of the multidisciplinary care.

** Key words:**Cystic fibrosis, oral health, teeth, United States.

## Introduction

Cystic Fibrosis (CF) is a multisystem autosomal recessive genetic disease (1). Treatments for CF include aggressive airway clearance therapy and nutrition support, often including regular high calorie snacks, nutritional supplements, and pancreatic enzyme replacement therapy (1). Chronic CF treatments often include recurrent antibiotics (i.e. for pulmonary exacerbations or sinusitis) and surgical procedures (such as sinus surgery, bronchoscopy, port-a-cath placements) (1).

Due to the high calorie (sugar) diet, sugar-containing medications, and the regular high calorie supplements, CF patients may be at risk for dental caries (2). Also, as a result of the effect of CF on the exocrine glands, low salivary flow and buffering capacity of CF children can additionally increase their caries risk (3). Most studies have concluded that CF children had lower caries prevalence than control children, whereas, few studies report increased caries risk in CF patients (4). Regarding oral hygiene, recent studies have suggested that plaque scores of CF children tend to be similar to healthy children (5,6) and that CF children have less gingival inflammation compared to controls (5,7,8).

In addition to diet and saliva, recurrent courses of antibiotics and multiple surgeries may have adverse effects on tooth development (9-11). There has been suggestion that Cystic Fibrosis Transmembrane Regulator (CFTR) function may directly affect enamel growth (12). Evidence suggests that metabolic insult in enamel development related to the CFTR leads to the enamel defects in the postnatal period. In the past, there was a higher prevalence of enamel hypoplasia and tooth discoloration as it related to tetracycline therapy for CF pulmonary exacerbations (13). However, current available alternative antibiotic regimens may have lowered their prevalence.

In the US, only three studies have reported oral manifestations in CF patients and all of them were conducted in the late 1970’s (13-15). Not only has the oral health status of the general population changed throughout the years, but the overall health, treatment, and survival of CF patients is drastically different from that time. Also, despite the oral health concerns and the multisystem approach to CF care, there are no established guidelines for CF patients’ dental care. Thus, the aim of the present study was to describe the oral health status of CF patients in a US facility to help health providers guide the oral health of these patients.

## Material and Methods

-Study Design

This was a cross sectional study that was in full accordance with the World Medical Association Declaration of Helsinki and had the approval of the Children’s Hospital of Wisconsin (CHW) Institutional Review Board.

-Sample

Data were collected from patients of the CHW CF Clinic. The inclusion criteria were children: a) 6-18 years old, b) with a diagnosis of CF. A total of 76 patients fulfilled the inclusion criteria. Exclusion criteria were children: a) with no permanent teeth present, and b) CF patients that had any other systemic disease with the exception of asthma. A total of 26 children fulfilled both the inclusion and exclusion criteria. Of them 20, with median 13 years of age and their parents, agreed to participate in the study and the response rate was 76.9%. Τhe power was calculated at 80.4% power with effect size=0.3 and α=0.05.

-Procedure

Parental informed consent was obtained for all patients and patient assent was obtained if patient was 7 years old and older.

A health questionnaire was filled out by a family member including: a) the types and age(s) he started inhaled treatments, b) if a child has ever taken antibiotics for longer than a week, and c) how many antibiotic courses, d) if the child has had any past CF-related surgeries, types and age they occurred, e) if there are any other pertinent health issues, illnesses, or diseases. In order to eliminate recall bias, health information given by the parents was cross-checked with their medical file. The health questionnaire also included a dental risk assessment querying a) if the child has a history of cavities, b) how often the child brushes his/her teeth, c) how often the child flosses his/her teeth, d) how often the child uses a fluoridated toothpaste, e) how often the child attends the dentist for regular check-ups, f) and how often the child eats a sugar containing snack or drink in between meals.

A clinical exam was then performed on each patient using a mirror and a ball ended probe. The clinical exam was a standard noninvasive dental exam with universal disinfection precautions. Two pediatric dentists performed the examinations. An examiner training and calibration comparing the examiners recording on 10 healthy patients was conducted prior to the initiation of the study. Inter-examiner and intra-examiner reliability was assessed using Cohen’s Kappa statistics and was κ=0.86 for inter-examiner agreement and κ=0.92 for intra-examiner agreement.

The following variables were recorded: i) dental caries using the dmft/DMFT index based on the International Caries Detection and Assessment System (ICDAS II)(16) criteria; ii) dental hygiene marking plaque levels using the Simplified Greene-Vermillion Dental Plaque Index (DI-S)(17); iii) gingival inflammation using the Community Periodontal Index of Treatment Needs (CPITN)(18); iv) and enamel defects using the modified Developmental Defects of Enamel Index (DDE) (19).

-Statistical Analysis

Descriptive statistics were used for the oral health status and behaviors of the sample by calculating Median and Interquartile Range or Mean and Standard Deviation. Further statistical analysis was performed using Fisher’s exact test to identify associations between dental caries and developmental dental defects with the type, time and number of antibiotic courses and surgeries CF patients received. Software (SPSS 16.0) was used to conduct these statistical analyses. Statistical significant differences were reported at the level of *p*<0.05. The findings were compared with published norms, where available.

## Results

-Questionnaire

The types of medications taken by CF children and the frequency of queried surgeries they had undergone are presented in  Table 1. There were no significant differences with the types of medications among CF patients. Results from the questionnaires ( Table 1) revealed that 40% of CF patients experienced past caries. The majority of parents of CF children (90%) reported that their children brush their teeth 1-2 times a day and 62.5% reported flossing only rarely. CF children had a sugar containing snack or drink more than twice a day 65% of the time. In addition, 70% of CF children attended regular dental check-ups every 6 months while the remaining 30% visited the dentist only once a year.

**Table 1:**
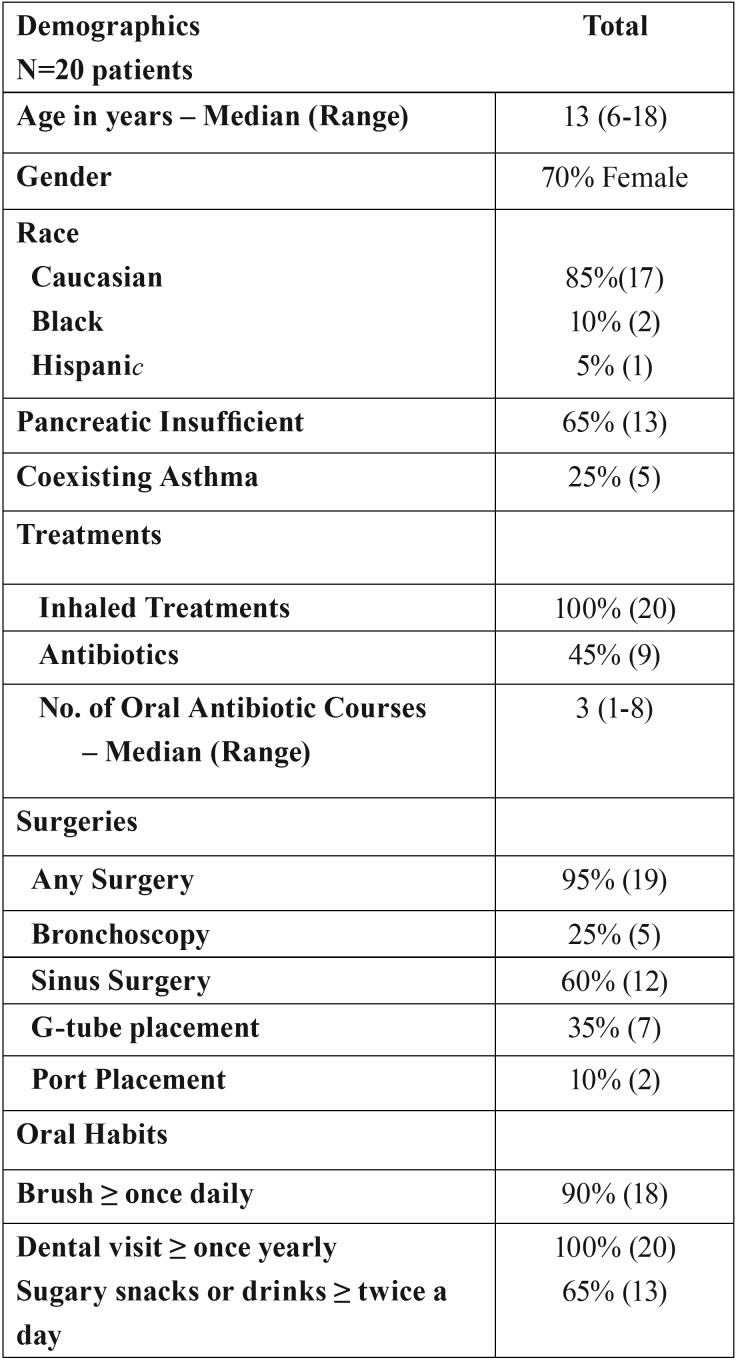
Demographics, history, and oral habits of included CF patients.

-Clinical Exam

**Figure 1:**
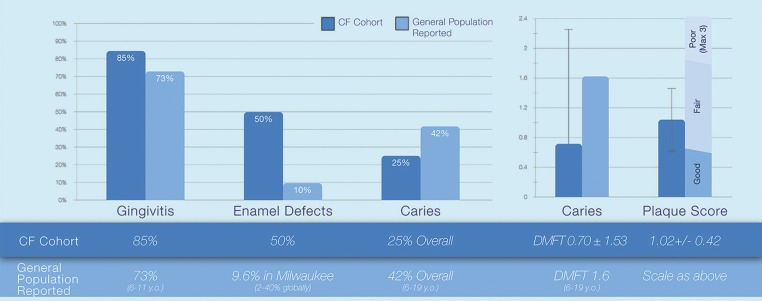
Prevalence of CF Patients’ dental issues versus reported general population.

The oral clinical findings of CF children are presented in Figure 1. DMFT was 0.25 ± 0.64 for the CF patients and dmft was 0.90 ± 2.02. When including patients with primary and permanent teeth, the combined index value is 0.70 ± 1.53. DMFT including incipient lesions was 0.50 ± 0.76 for CF patients. As for enamel defects, 50% of subjects had enamel defects on their permanent teeth with 60% of them having 1st permanent molars and incisors affected. More specifically, 10% had a demarcated opacity, 30% a diffuse opacity and 10% had enamel hypoplasia. Plaque score was 1.02 ± 0.42, which is considered fair oral hygiene. Lastly, 85% of CF patients had gingivitis. More specifically, 75% presented gingival bleeding after gentle probing with no periodontal pockets, calculus, or overhanging restorations categorized as CPTIN=1. Also, 10% had periodontal pockets less than 3mm and supra gingival calculus (CPTIN=2).

-Associations

The age that any type of medication was started, either inhaled treatments or antibiotics, did not show a significant effect on the type of tooth with an enamel defect. However, a significant correlation was found between the number of courses of antibiotics taken by CF children and the number of teeth affected with enamel defects (*p*=0.007) with the children with more antibiotics having more enamel defects. No significant difference existed between the type of medication used and the severity of enamel defects. CF children taking antibiotics had a trend towards more severe enamel defects (*p*= 0.017). As for dental caries, the type of medication did not show any statistically significant difference (*p*=0.802).

No differences were found between CF children with and without asthma. More specifically, children did not differ significantly in the presence of dental caries (*p*=0.339), the presence of dental defects (*p*=0.606), dental hygiene (*p*=0.186), or gingivitis (*p*=0.670).

The type and time of surgeries these patients had, showed no statistically significant differences to the severity of enamel defects presented. However, there was a trend, indicating that the more surgeries the CF child had, the more severe enamel defects they presented (*p*=0.076). Lastly, it was found that an increase in number of surgeries CF children had was correlated with an increase in dental caries (*p*=0.028).

## Discussion

Within the limitations of the present study, results suggest that despite the high frequency of sugary snacks in the diet of CF children, there is a relatively low rate of dental caries. Also, patients seem to have fair oral hygiene and all visit the dentist frequently. However, the frequency of enamel defects was found to be much more worrisome, with the severity thereof increasing with the number of antibiotic courses taken and the number of surgeries they had undergone.

The sample of this study was chosen from the CHW CF Center. The previous US studies had a larger sample varying from 42 to 86 CF patients (13-15). However, all these studies were conducted in the 70s-80s when research compliance was less complicated allowing higher participation rate. This specific age group was selected based on the beginning of the early transitioning to adult dentition at age six. CF disease and its treatments could potentially influence permanent teeth formation after birth as these teeth continue to develop and calcify (20). Primary teeth have already developed in utero at this point, and therefore the effect to these teeth is minimal. CF patients with other systemic diseases were excluded in order to minimize the risk of detecting oral health effects related to these diseases (21). Asthmatic CF patients were included in the study, but no difference between asthmatic and non-asthmatic children’s oral health was detected. The oral status and CF history was assessed through a questionnaire and clinical examination in an indices based quantitative manner, both considered simple and reliable (22).

As expected, CF children are encouraged to eat more frequently and often consume foods with high sugar contents such as juice, milk, nutritional supplements, and so forth, which are known to result in high caries prevalence (9). High cariogenic diet has also been reported in previous studies (15,23). Proper oral hygiene can prevent the formation of caries and mitigate some of this risk. Despite the high consumption of cariogenic foods, we found a lower rate of caries in the CF children than in the general population. Additionally the correlation between frequency of sugary snacks/drinks and caries was not statistically significant, which is in accordance with previous studies (23). Their fair oral hygiene and frequent visits to the dentist likely contributed to this finding. Similar results have been reported in past studies, with twice a day brushing ranging from 60% to 90% (5,23,24). Frequent application of oral hygiene in CF children has been attributed to their parents increased awareness and motivation of proper health(24). In addition, 70% of our patients attended regular dental check-ups every 6 months. According to a recent study, CF patients lack a dental home in comparison to healthy children (25). However, the participants of our study had access to dental care regularly. This may be in part due to parents’ awareness on the importance of oral health and chronic disease care.

Clinically, CF children had lower caries prevalence in comparison to the national DMFT mean of 1.6 for children 6-19 years old in USA (Fig. 1) (26). Our finding in a US CF Center is in accordance with the majority of the literature that suggests a lower caries risk among CF patients in comparison to healthy controls, both historically (13,14) and internationally (4,7,8,23,24). The low caries prevalence found in CF children is usually attributed to diligent oral hygiene, frequent dental visits, and use of antibiotics (2). The caries prevalence presented here is even lower than the previous US studies (13,14). In those studies, dmft was 2.6 and 5.5, and DMFT was 7.5 and 7.9 whereas the present study found dmft of 0.90 and DMFT of 0.25. This is again multifactorial as caries prevalence has declined globally through time (26) and CF care and survival has drastically changed.

CF children had fair oral hygiene that did not differ from the national mean of 1.42(27) (Fig. 1). Interestingly, oral hygiene was similar to the previously US reported score among CF patients of 0.90 in the 1970s(14). This finding is in accordance to recent studies that report similar plaque scores between healthy and CF children (5,6). However, in the past, studies have reported a tendency for lower plaque scores among CF children (8,14,15). Regarding gingival health 75% of CF children had mild gingivitis which is lower than the national mean of 85%(28) (Fig. 1) and is in accordance with the majority of other studies that have also suggested less gingival inflammation among CF children (5,7,8,15). The percentage of gingivitis was similar to a previous US study of CF patients that found mild gingivitis in 71% of the patients (15).

Moreover, 50% of the CF children had some enamel defects whereas the prevalence of similar defects in healthy children was around 2-40% (Fig. 1) (29). Although findings in CF patients have varied, most European or historical case-control studies have found higher prevalence of enamel defects among CF patients in comparison to healthy controls (10,11,13,14,24). Studies in the past have shown that the high number of surgeries and medications, mainly the high intake of antibiotics, lead to more enamel defects on permanent teeth in CF patients (13). Interestingly the percentage of enamel defects reported in previous US studies was relatively low in comparison to what we found here (13,14). The exact reason for this increase is unclear however it may be affected by current courses of medication, surgical needs, and more aggressive CF care.

This study showed that there was no significant difference between the types of medications and surgeries CF patients had. Due to the small sample size, and the multiple options for medications and surgeries by each CF individual, no clinical significance was found. The most significant correlation we found was between the number of antibiotics and both the frequency and severity of enamel defects. This likely suggests that the antibiotics contribute to an insult in the enamel during the tooth calcification stage. Also, results from this study found that there was a trend of number of surgeries to correlate with the severity of enamel defects, and a statistical significance with the number of surgeries and caries prevalence for CF children. These findings suggest that multiple surgeries on a child in his/her early years may have an effect towards tooth calcification, hence leading to a higher risk for caries and a trend towards more severe enamel defects.

The small sample size of only twenty CF children is one of the limitations of this study. Recruitment of CF patients was challenging at times and families were not always interested in extending their visit to the clinic. Another limitation is the large age range which increased due to difficulty in recruitment. Further studies are required with a larger sample size and likely multicenter, to further ascertain the significance and prevalence of these findings, as well as association with other aspects of CF disease.

Overall, within the limitations of this pilot study, we found that despite the high calorie diet of CF patients there was not an increase in dental caries. However, a significant amount of enamel defects were found. This finding is concerning as enamel defects can lead to aesthetic issues, tooth sensitivity, and loss of tooth structure, which can beget pain, need for restorative treatments (ie crowns), or eventual tooth loss. Advanced restorative techniques may be required to rehabilitate teeth with severe enamel defects, and weakened tooth structure.

As CF patients continue to reach farther into adulthood, these problems can have longer lasting or more significant effects on the health of CF patients. Further large studies are needed to determine the dental needs and full risk for these patients. However, clearly regular dental care should be a part of routine CF counseling, as they are at risk for oral health issues. Results such as of this study can help to illustrate the importance of regular dental care, especially as the need for aggressive treatments generally increases as CF patients age. Children with CF, as all children, should brush twice a day, floss regularly, and see a dentist yearly (30). With an established dental home, CF patients can be carefully monitored at every visit to ensure proper oral hygiene, and necessary restorative dental care, and thus prevent further dental issues in the CF population.
